# Sensitive and Selective Detection of Tartrazine Based on TiO_2_-Electrochemically Reduced Graphene Oxide Composite-Modified Electrodes

**DOI:** 10.3390/s18061911

**Published:** 2018-06-12

**Authors:** Quanguo He, Jun Liu, Xiaopeng Liu, Guangli Li, Peihong Deng, Jing Liang, Dongchu Chen

**Affiliations:** 1 School of Materials Science and Energy Engineering, Foshan University, Foshan 528000, China; hequanguo@hut.edu.cn (Q.H.); junliu@hut.edu.cn (J.L.); guangli010@hut.edu.cn (G.L.); 2 Department of Chemistry and Material Science, Hengyang Normal University, Hengyang 421008, China; amituo321@163.com (X.L.); liangjingabbey@126.com (J.L.); 3 Hunan Key Laboratory of Biomedical Nanomaterials and Devices, College of Life Sciences and Chemistry, Hunan University of Technology, Zhuzhou 412007, China

**Keywords:** titanium dioxide, reduced graphene oxide, modified electrode, tartrazine, electrocatalysis

## Abstract

TiO_2_-reduced graphene oxide composite-modified glassy carbon electrodes (TiO_2_–ErGO–GCE) for the sensitive detection of tartrazine were prepared by drop casting followed by electrochemical reduction. The as-prepared material was characterized by transmission electron microscopy (TEM) and X-ray diffraction (XRD). Cyclic voltammetry and second-order derivative linear scan voltammetry were performed to analyze the electrochemical sensing of tartrazine on different electrodes. The determination conditions (including pH, accumulation potential, and accumulation time) were optimized systematically. The results showed that the TiO_2_–ErGO composites increased the electrochemical active area of the electrode and enhanced the electrochemical responses to tartrazine significantly. Under the optimum detection conditions, the peak current was found to be linear for tartrazine concentrations in the range of 2.0 × 10^−8^–2.0 × 10^−5^ mol/L, with a lower detection limit of 8.0 × 10^−9^ mol/L (S/N = 3). Finally, the proposed TiO_2_–ErGO–GCEs were successfully applied for the detection of trace tartrazine in carbonated beverage samples.

## 1. Introduction

With the rapid development of modern life, artificially sweetened beverages attract a lot of children because of their colorful appearance and sweet taste. Food colorants are commonly used in these artificially sweetened beverages as additives. Tartrazine is an important food colorant used in various kinds of drinks and foods [1,2]. However, the azodi group and aromatic ring in tartrazine are found to be a potential risk to human health; thus, the dosage of tartrazine used should be monitored constantly. The acceptable daily intake (ADI) of tartrazine recommended by the FDA is 3.75 mg/kg for humans. Moreover, the World Health Organization (WHO) suggest that the ADI could be limited to 2.5 mg/kg. The excessive intake of tartrazine could cause various diseases, such as allergy, asthma, eczema, anxiety, migraine, and even cancer [3,4]. More importantly, the accidental abuse of tartrazine (such as from tainted steamed buns) has often happened in recent years. Thus, detecting the content of tartrazine in foods and drinks is important for improving food security in the modern society. Developing a detecting method characterized by celerity, high sensitivity, and high selectivity is a significant way to promote the progress of human health.

In recent years, various methods have been developed for detecting the content of food colorants [5,6,7], especially for the detection of tartrazine, such as general fluorescent methods [8,9], enzyme-linked immunosorbent methods [10], and electroanalytical methods [11]. The fluorescent method requires that the analyzed materials possess fluorescent properties, which restricts its application. The enzyme-linked immunosorbent method is a novel biological technique. It requires expensive biological materials, such as antibodies, and its complex operation steps could cause inaccuracy in the colorant detection. The electroanalytical method is one of the most important ways for trace detection and possesses various advantages, such as low cost, high sensitivity, and high selectivity [12]. Various modified electrodes have been developed for the electrochemical detection of tartrazine. For example, Squella and co-workers prepared multi-walled carbon nanotube (MWCNT)-modified GCE by using 1,3-dioxolane as a dispersant agent. The proposed sensor was successfully applied to the simultaneous detection of tartrazine, sunset yellow, and carnosine with high sensitivity and reproducibility. The sensitivity (3.5 μA/μM) toward tartrazine was improved significantly with a low limit of detection (0.22 μM), due to the large surface area and high electrical conductibility of the MWCNTs [13]. Multi-walled carbon nanotubes–film-coated glassy carbon electrodes have also been proposed to determinate tartrazine, and successfully applied to determinate tartrazine in soft drinks [14]. Gold nanorods–graphene-modified electrodes were developed for the sensitive determination of tartrazine, and exhibited a linear response in the concentration range of 0.03–6.0 μM, with a detection limit of 8.6 nM [15]. A novel electrochemical sensor based on ionic liquid-modified expanded graphite paste electrode showed excellent sensing performance, including a wide linear response range (0.01 μM–2 μM), low detection limit (3.0 nM), good reproducibility, stability, and reusability [16]. Although these modified electrodes exhibit outstanding sensing performance towards tartrazine, their high costs make them commercially unfavorable. Besides, the stability of the ionic liquid-modified electrode is poor, since ionic liquid is susceptible to the air moisture. Hence, it is necessary to seek suitable electrode modification materials with low cost, excellent electrocatalytic performance, large surface areas, and excellent electrical conductibility. 

It is well known that carbon-based nanomaterials possess various advantages, such as low cost, good electrical conductibility, and high surface area and have been developed intensely. These carbon materials mainly include carbon nanofibers, porous activated carbon, carbon nanotubes, fullerene, and graphene. Graphene, an emerging 2-dimensional (2D) nanomaterial, has been widely applied in many fields, such as materials science, biomedicine, aerospace, and electronics, owing to its outstanding merits, including good thermal and electron conductivity, high number of surface active sites, and low-cost raw materials [17,18]. In the field of electrochemical analysis, graphene is regarded as one of the preferred candidates for modified electrodes. In our previous reports, a series of metal oxides–graphene nanocomposite-modified GCEs have been developed for the sensitive detection of dopamine and vanillin [19,20,21]. In these nanocomposite systems, various metal oxides, used as the main functional materials, such as Fe_3_O_4_, Fe_2_O_3_, MnO_2_, and Cu_2_O, show good electrocatalytic activities in electrochemical analyses [22,23,24,25]. The detection sensitivity was enhanced greatly because these semiconductors offer more active reaction sites for the analyses. TiO_2_, as a common semiconductor, has been developed for several decades. TiO_2_ nanomaterials are widely used in the photocatalytic field, because of their good catalytic activity [26,27,28]. Recently, the electrocatalytic activity of TiO_2_ has also been reported. For example, Arkan and co-workers fabricated a TiO_2_ nanoparticles–carbon nanofiber-modified GCE, and the results demonstrated that the proposed modified electrode lowered the overpotential of idarubicin (IDA) during the oxidation process. The composite exhibited a good electrocatalytic activity with a wide liner range from 0.012 to 10 μM [29]. 

By coupling the merits of 2D graphene materials (larger surface area and high electrical conductivity) with the good catalytic performance of TiO_2_ semiconductors, the electrochemical properties may be improved for the detection of trace food colorants. However, to the best of our knowledge, TiO_2_–graphene nanocomposite-modified electrodes for tartrazine have rarely been reported. A novel electrochemical sensor based on graphene–mesoporous TiO_2_-modified carbon paste electrodes was developed for the detection of trace tartrazine and exhibited a wide linear detection range (0.02–0.18 μM) and a low detection limit (8.0 nM) using square wave voltammetry (SWV). However, the graphene was obtained by chemically reduction of graphene oxide, which requires a poisonous reductant and several steps in the reduction process. Herein, TiO_2_-electrochemically reduced graphene oxide-modified glassy carbon electrodes (TiO_2_–ErGO–GCEs) were prepared by a facile hydrothermal and electrochemical reduction method. Second-order derivative linear sweep voltammetry was employed to detect tartrazine, because of its advantages, including higher sensitivity and better selectivity than differential pulse voltammetry (DPV) and square wave voltammetry (SWV) [30]. The morphologies and structures of these samples were analyzed by transmission electron microscopy (TEM) and powder X-ray diffraction (XRD). Moreover, the electrochemical behavior of tartrazine on TiO_2_–ErGO–GCE was investigated in detail. Furthermore, various electrochemical parameters (pH, scan rate, accumulation potential, and time) were discussed. Finally, the TiO_2_–ErGO–GCE was successfully applied for tartrazine detection in a carbonate beverage.

## 2. Experimental Section

### 2.1. Materials and Chemicals

Titanous sulfate (Ti(SO_4_)_2_), graphite powder, sodium nitrate (NaNO_3_), concentrated sulfuric acid (H_2_SO_4_), potassium permanganate (KMnO_4_), hydrogen peroxide (H_2_O_2_), potassium nitrate (KNO_3_), phosphoric acid (H_3_PO_4_), sodium hydroxide (NaOH), hydrochloric acid (HCl), and ethyl alcohol were purchased from Sinopharm Chemical Reagent Co., Ltd. (Shanghai, China). Tartrazine was purchased from Sigma-Aldrich Co. (Shanghai, China). All reagents were used without further purification. Finally, ultrapure water was used in all experiments (18.2 MΩ).

### 2.2. Synthesis of TiO_2_ Nanoparticles (NPs)

The TiO_2_ NPs were synthesized according to a published method [31]. Typically, 4.899 g of Ti(SO_4_)_2_ were dissolved in 50 mL of water under stirring for 30 min. This solution was transferred to a Teflon-lined stainless-steel autoclave (100 mL) and reacted at 200 °C for 4 h. After cooling to room temperature, the TiO_2_ reactants were centrifugated at 7920 rcf. Then, the samples were washed with water and ethyl alcohol for several times, and the TiO_2_ NPs were obtained by drying at 60 °C in vacuum for 10 h.

### 2.3. Synthesis of TiO_2_–GO Composite Nanomaterials

Graphene oxide (GO) was synthesized by the modified Hummers’ method [32]. In a typical process, concentrated H_2_SO_4_ was cooled down to 0 °C, and 0.5 g of graphite powder and NaNO_3_ were added subsequently under stirring. Then, 3.0 g of KMnO_4_ was added slowly at 5 °C. After that, the temperature was raised to 35 °C for 2 h under stirring to form a mash, and 40 mL of water was added at 50 °C; then, the temperature was increased to 95 °C for 0.5 h. The above solution was added to 20 mL of 30% H_2_O_2_ in batches. The as-obtained precipitate was washed with 150 mL of hydrochloric acid (1:10) and 150 mL of H_2_O. Then, it was vacuum-dried at 50 °C for 12 h to obtain the graphite oxide. Subsequently, 100 mg of GO were dispersed in 100 mL of water under ultrasound exfoliation for 2 h. Finally, 2 mg of TiO_2_ NPs was added to 5 mL of GO supernatant solution (1 mg/mL) under ultrasounds for 2 h to obtain the TiO_2_–GO composite.

### 2.4. Fabrication of TiO_2_–ErGO-Modified GCE

Firstly, the polished GCE was immersed in ethyl alcohol and water under ultrasounds for 1 min. Then the TiO_2–_GO–GCEs were fabricated via drop casting of the TiO_2_–GO dispersion on the GCE, followed by an electrochemical reduction process. Specifically, 5 μL of TiO_2_–GO dispersion was dropped and casted on the surface of bare GCE to prepare the TiO_2_–GO–GCE. Then, the TiO_2_–GO–GCE was reduced by electrochemical reduction under the potential of −1.2 V for 120 s for the formation of TiO_2_–ErGO–GCE. Reduced graphene oxide-modified GCEs (ErGO–GCE) were also prepared for comparison.

### 2.5. Characterization

TEM images were obtained by JEOL JEM-2010 (HT, Tokyo, Japan), operated at 200 kV. XRD patterns were obtained by X-ray diffractometry (PANalytical, Holland), performed at 40 kV and 40 mA with Cu Kα radiation (λ = 0.1542 nm). 

### 2.6. Electrochemical Experiments

Both cyclic voltammetry (CV) and second-order derivative linear sweep voltammetry (SDLSV) were carried out with a standard three-electrode system. The bare or nanomaterial-modified GCEs were used as working electrodes. A platinum electrode and a saturated calomel electrode (SCE) acted as counter electrode and reference electrode, respectively. The CVs were measured by CHI 660E electrochemical workstation (Chenhua Instrument Co. Ltd., Shanghai, China), and the SDLSV was tested by a JP-303E Polarographic Analyzer (Chengdu Instrument Company, Chengdu, China). Fresh PBS, 0.1 M, was used as a supporting electrolyte for all electrochemical tests. Unless stated otherwise, all electrochemical tests were recorded at a scan rate of 100 mV/s, after a suitable accumulation period under stirring at 500 rpm and a 5 s rest. The potential scan ranges were 0.4–1.2 V for CV and 0.6–1.2 V for the SDLSV.

### 2.7. Analysis of Real Samples

The carbonate beverage was purchased from a local supermarket. The CO_2_ was eliminated by an ultrasound process. An amount of 1 mL of sample was diluted to 6 mL with 1.0 M PBS (pH 3.7). Then, carbonate beverage samples at various concentration were prepared by dilution. The content of tartrazine in the carbonate beverage was measured using SDLSV by the standard addition method under the optimal detection conditions.

## 3. Result and Discussion

### 3.1. Morphologic and Structural Characterization of TiO_2_–GO Nanocomposites

**Figure 1:**
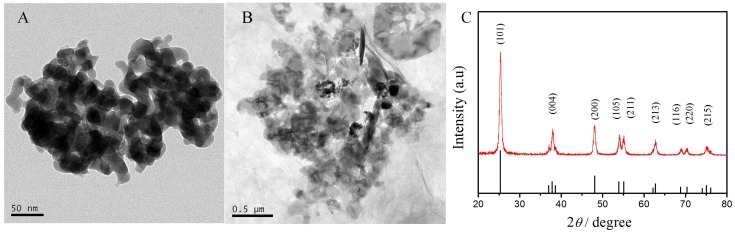
The TEM images of TiO_2_ (**A**) and TiO_2_–graphene oxide (GO) (**B**); The XRD pattern of TiO_2_ nanoparticles (NPs) (**C**).

The morphologies of the TiO_2_ and TiO_2_–GO composite samples were characterized by TEM. As shown in the TEM images (Figure 1A), the TiO_2_ NPs was cube-like with an average particle size of 50 nm. The TiO_2_ NPs aggregated with each other, and their dispersibility could be improved. Moreover, GO sheets were obviously observed in the surrounding of the TiO_2_ NPs (Figure 1B), indicating that the TiO_2_ NPs were well combined with the GO nanosheets. The XRD pattern of the TiO_2_ NPs is also presented (Figure 1C). The standard JCPDS card of pure anatase TiO_2_ (21-1272, black lines) were used for comparison. The apparent diffraction peaks of anatase TiO_2_ located at 25.37, 37.03, 37.88, 48.12, 53.97, 55.10, 62.14, 68.78, 70.35, and 75.13 could be indexed to (101), (004), (200), (105), (211), (213), (116), (220), and (215) planes of anatase TiO_2_. This indicates that as-prepared TiO_2_ NPs presented the anatase structure with high crystallinity. 

### 3.2. Electrochemical Behavior of Tartrazine on Different Electrodes

**Figure 2:**
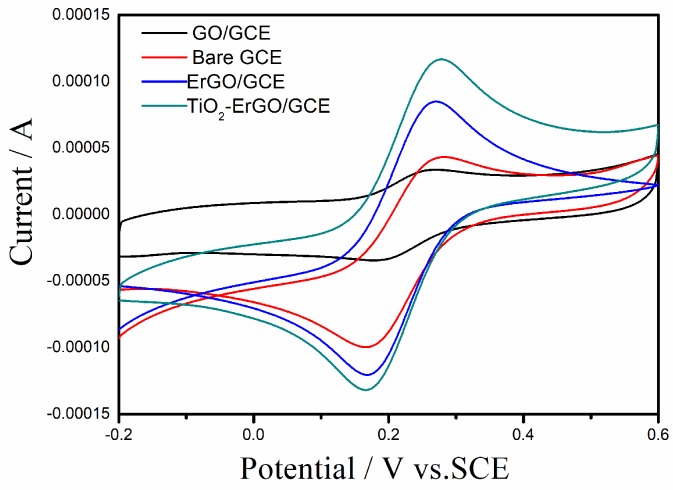
Cyclic voltammograms of bare glassy carbon electrode (GCE), GO–GCE, reduced graphene oxide-modified GCEs (ErGO–GCE), andTiO_2_–ErGO–GCE in 2.5 × 10^−3^ mol/L [Fe(CN)_6_]^3−/4−^ solution.

The cyclic voltammograms of bare GCE, GO–GCE, ErGO–GCE, andTiO_2_–ErGO–GCE in 2.5 × 10^−3^ mol/L [Fe(CN)_6_]^3−/4−^ solution were investigated, and the results are shown in Figure 2. As expected, a pair of reversible redox peaks appeared on all electrodes. However, the intensities of the redox peaks increased in the following order, GO–GCE, bare GCE, ErGO–GCE, and TiO_2_–ErGO–GCE. The redox peak current on the GO–GCE was the smallest because of the poor electrical conductivity. When GO was reduced to ErGO, the redox peak current enhanced greatly because of the restoration of the conductive carbon conjugate network and large surface area. When ErGO was combined with TiO_2_ NPs, the redox peak current further increased because of a synergistic enhancement between ErGO and TiO_2_ NPs. The reduction peak currents of bare GO, GCE, RGO–GCE, and TiO_2_–RGO–GCE were 3.37 × 10^−5^ A, 4.32 × 10^−5^ A, 8.49 × 10^−5^ A, and 1.17 × 10^−4^ A, respectively. According to Randles–Sevcik equation, their electroactive area were estimated as 0.058 cm^2^, 0.074 cm^2^, 0.145 cm^2^, and 0.199 cm^2^, respectively. The electrochemical active area of bare GCE coincided with the geometric area (Φ 3.0 mm, 0.071 cm^2^), and the electrochemical active area of ErGO–CCE and TiO_2_–ErGO–GCE were approximately 2.0 and 2.7 times that on the bare GCE, in relation to the large specific surface area of TiO_2_ NPs and ErGO. The large electrochemical active area of the TiO_2_–ErGO nanocomposites will enhance the adsorption capacity of tartrazine and offer more catalytic sites for tartrazine oxidation.

**Figure 3:**
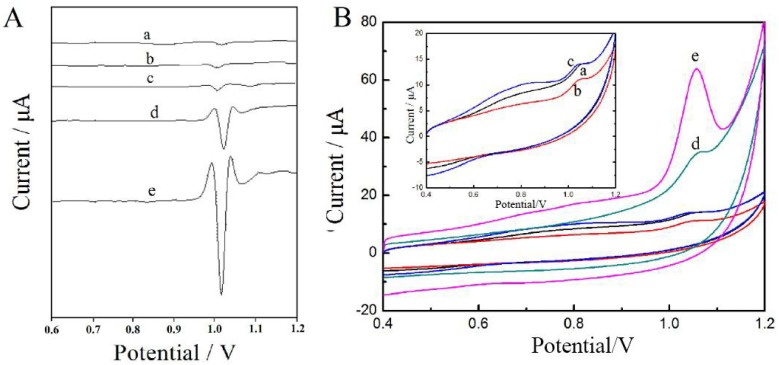
Second-order derivative linear sweep voltammetry (SDLSV) (**A**) and cyclic voltammetry (CV) (**B**) of 1.0 × 10^−5^ mol/L tartrazine on different electrodes (a: GCE; b: GO–GCE; c: TiO_2_–GO–GCE; d: ErGO–GCE; e: TiO_2_–ErGO–GCE).

The electrochemical behavior of tartrazine on (1.0 × 10^−5^ mol/L) the surface of the bare and modified GCEs were investigated by SDLSV. The results are shown in Figure 3A. A wide and short peak appeared on the surface of the GCE at 1000 mV (curve a), and the peak current was 1.594 μA. The peak current of tartrazine on the surface of GO–GCE was 1.006 μA (curve b); the lower current could be attributed to the inferior electrical conductivity of GO. Moreover, a wide and short peak (1004 mV, 1.987 μA) were detected on the surface of TiO_2_–GO–GCE, probably because of the catalytic properties of TiO_2_ with mesoporous structure and the poor electrical conductivity of GO. However, a manifest oxidation peak located at 1036 mV was observed on the ErGO–GCE, and the peak current increased significantly to 20.10 μA (curve d). This phenomenon could be ascribed to the higher electrical conductivity of ErGO due to the restoration of the conductive carbon-conjugated structure. Moreover, the large surface area could promote the adsorption of tartrazine onto the electrodes. More importantly, the peak current further increases to 26.98 μA when TiO_2_–ErGO-GCE acted as the work electrode (curve e). The peak current on the TiO_2_–ErGO–GCE was 18 times higher than that on the bare GCE, because of the synergistic effects of TiO_2_ and ErGO that enhanced the electrochemical oxidation of tartrazine. 

The CV curves of bare and modified GCEs recorded in 1.0 × 10^−5^ mol/L of tartrazine solution are presented in Figure 3B. Only the oxidation peak can be observed on all the electrodes, suggesting that the electrochemical oxidation of tartrazine is an irreversible process. The order of peak current for the different electrodes was consistent with the results of SDSLV. As expected, the largest peak current was obtained on the TiO_2_–ErGO–GCE, further confirming that the synergistic effect of TiO_2_ and ErGO improved the electrochemical oxidation of tartrazine. 

### 3.3. Optimization of the Detection Conditions of Tartrazine

#### 3.3.1. The Influence of pH

**Figure 4:**
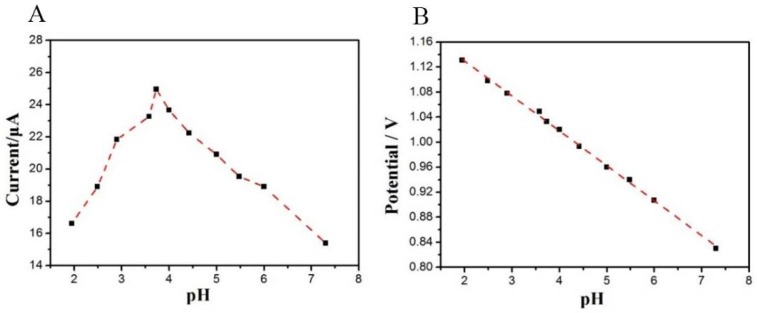
(**A**) Influence of pH on the oxidation peak currents in a 1.0 × 10^−5^ mol/L tartrazine solution on the TiO_2_–ErGO–GCE; (**B**) The linear plot of tartrazine oxidation peak potential and pH.

Since the pH is an important parameter influencing the electrochemical oxidation of tartrazine, it is important to evaluate the optimal pH value for tartrazine detection. As shown in Figure 4A, the largest current intensity (*i_pa_*) of tartrazine was observed when the pH was 3.7. The electro-oxidation of tartrazine was performed better in more acidic media, whereas in neutral to alkaline media, the anodic peak current was considerably decreased. Moreover, the oxidation peak potential *E_p_* is linear to the pH in the pH range of 2.5–7.5 (Figure 4B). The linear equation was *E_p_* = −0.0560 *pH* + 1.024 (R^2^ = 0.999), and the slope (−63 mV/pH) was very close to the theoretical value (−59 mV/pH)**,** indicating that the same electron and proton number participate in the electrochemical oxidation process.

#### 3.3.2. Effect of Accumulation Conditions

**Figure 5:**
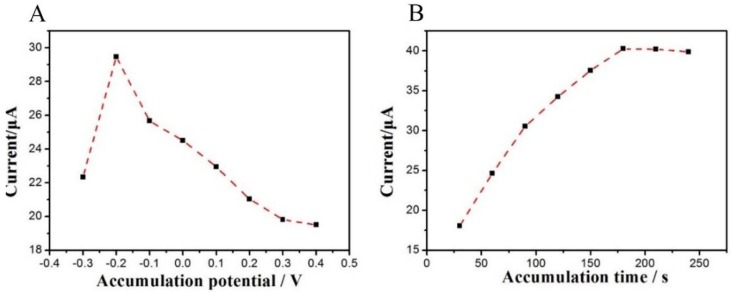
Effects of accumulation potential (**A**) and accumulation time (**B**) on the oxidation peak currents of 1.0 × 10^−5^ mol/L Tartrazine at TiO_2_–ErGO–GCE.

Accumulation potential and time are other two important factors that influence the oxidation current of tartrazine. After accumulation for 180 s with different accumulation potentials (−0.3 to 0.4 V), the oxidation peak currents in 1 × 10^−5^ mol/L tartrazine were measured. When the accumulation potential was −0.2 V, the largest oxidation peak current was obtained (Figure 5A), indicating that −0.2 V was the best accumulation potential. In addition, the accumulation at −0.2 V for various times was also investigated. As plotted in Figure 5B, the oxidation peak currents increased with the accumulation time between 0 and 180 s. Afterward, the *i*_pa_ remained stable because of the saturation adsorption of tartrazine on the surface of TiO_2_–ErGO–GCE. Thus, 180 s was the optimal accumulation time.

#### 3.3.3. The Influence of the Scan Rate

**Figure 6:**
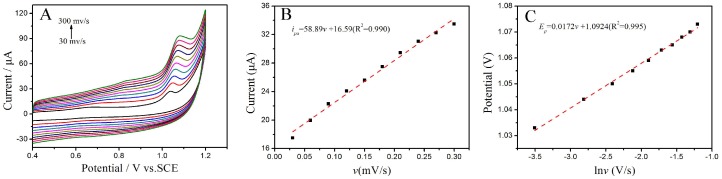
(**A**) CVs of 1.0 × 10^−5^ mol/L tartrazine on the TiO_2_–ErGO–GCE at different scan rates; (**B**) Relationship between oxidation peak current and scan rate; (**C**) Relationship between peak potential and the Napierian logarithm of the scan rate. Saturated calomel electrode (SCE).

The electrochemical response of tartrazine is strongly dependent on the scan rate, thus this parameter was also considered. The CVs were scanned at different scan rates (30~300 mV/s) in PBS (0.1 M, pH 3.7) solution containing 1 × 10^−5^ mol/L of tartrazine, and the results are presented in Figure 6A. The oxidation peak current increased gradually with the increase of the scan rate. As shown in Figure 6B, a good linear relationship between oxidation peak currents (*i_pa_*) and scan rate (*v*) was obtained, and the corresponding linear equation was *i_pa_* = 58.89*v* + 16.59 (R^2^ = 0.990). This result indicates that the electrochemical oxidation of tartrazine was an adsorption-controlled process. Thus, the accumulation method was adopted in the subsequent experiments in order to enhance the sensitivity. However, the background currents were also increased correspondingly. Considering the best signal to noise ratio (SNR) and the lowest background current, a suitable scan rate was found to be 100 mV/s. 

**Figure 7:**
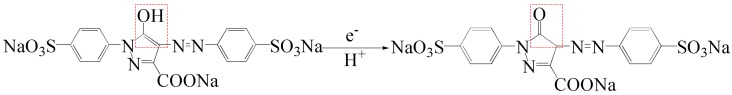
The mechanism of the electrochemical oxidation of tartrazine on the TiO_2_–ErGO–GCE.

Furthermore, only a positive oxidation peak potential (*E_pa_*) shifts with the rising of the scan rates (*v*) was observed, meaning that the oxidation process of tartrazine was irreversible. The liner relationship between *E_pa_* and the Napierian logarithm of the scan rate (ln *v*) is also presented (Figure 6C). The linear equation was *E_pa_* = 0.0172 ln *v* + 1.0924 (R^2^ = 0.995). According to Lavrion equation [33],

(1) $$\begin{array}{cc} E_{p} & =E^{0^{\prime }}-\frac{RT}{\alpha nF}[0.780+\ln(\frac{D^{1/2}}{k^{0}})+\ln{\left(\frac{\alpha nFv}{RT}\right)}^{1/2}]\\ & =K+\frac{RT}{2\alpha nF}\ln v \end{array}$$

where *E*^0′^ is the formal potential (V), *α* is the charge transfer coefficient, *n* is the electron transfer number, *F* is the Faraday constant (96,480 C·mol^−1^), *R* is the ideal gas constant (8.314 J·mol^−1^·K^−1^), *T* is the Kelvin temperature (*K*), *D* is the diffusion coefficient, and *k*^0^ is the heterogeneous electron transfer rate. The value of *α* is always supposed to be 0.5 in an irreversible process, and the *n* value is calculated as 1. Thus, the oxidation of tartrazine is an irreversible process with one electron and one proton. This result is consistent with those of a previous report [34]. The electrochemical oxidation mechanism of tartrazine is summarized in Figure 7. 

#### 3.3.4. Calibration Curve and Detection Limit

**Figure 8:**
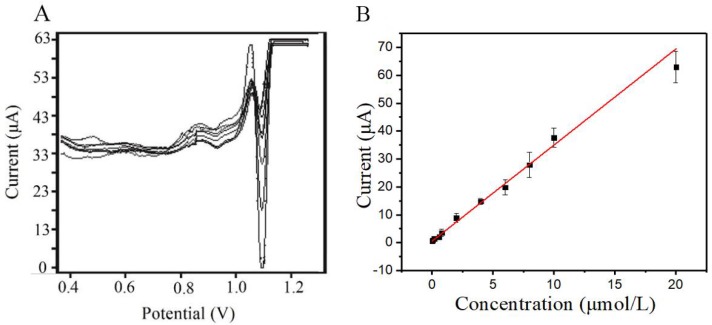
(**A**) SDLSV of different concentrations of tartrazine on the TiO_2_–ErGO–GCE; linear curve representing the relationship between oxidation peak current and concentrations of tartrazine in the range of 2.0 × 10^−8^–2.0 × 10^−5^ mol/L (**B**).

Under the optimal detection conditions, the SDLSV response of tartrazine at various concentration (range of 2.0 × 10^−8^–2.0 × 10^−5^ mol/L) was measured, and the results are presented in Figure 8A. With the increase of tartrazine concentration, the peak currents *i_pa_* were enhanced linearly. Moreover, the linear relationship between the peak currents *i_pa_* and the concentration of tartrazine was calculated as *i_pa_* (μA) = 3.450*c* (μmol/L) + 0.486 (R^2^ = 0.991, with a standard error of slope: 0.104 and standard error of intercept: 0.0704) (Figure 8B); the detection limit (S/N = 3) was calculated as 8.0 × 10^−9^ mol/L. This results were comparable and even better than those reported in the literature [35,36].

### 3.4. Interference Studies

The interference studies on species and other food colorants coexistent with tartrazine was also investigated. Different kinds of interfering species were added into 0.45 mol/L PBS (pH 3.7) containing 1.0 × 10^−5^ mol/L of tartrazine, and the peak currents were tested and compared. The peak currents *i_pa_* of 10 μmol/L of tartrazine in the presence of interferents are listed in Table 1. Under an acceptable error range, 100-times higher (than the concentration of tartrazine) concentrations of glucose, benzoic acid, citric acid, Na^+^, K^+^, and Fe^3+^, and 10-times higher concentrations of sunset yellow and amaranth did not interfere with the detection of tartrazine. Moreover, the peak currents intensities of tartrazine under the influence of interferents were still similar to those of pure Ttrtrazine, meaning that no oxidation peaks of the interfering species appeared or that the oxidation peaks separated well from those of tartrazine. This indicates that the TiO_2_–ErGO–GCE showed superior anti-interference performance and presents great prospects for the detection of tartrazine in various real samples. It is noteworthy that the oxidation peaks of sunset yellow and amaranth did not overlay with those of tartrazine, in spite of their similar structure. Hence, the TiO_2_–ErGO–GCE shows great potential for the simultaneous detection of tartrazine, sunset yellow, and amaranth. This work will be carried out in the future.

### 3.5. Reproducibility of the Detection

Under the optimal detection conditions, tartrazine standard solutions (1.0 × 10*^−^*^5^ mol/L) were detected for seven times by using the same TiO_2_–ErGO–GCE as the work electrode. After every test, the electrode was washed in 0.1 mol/L of nitric acid for two times under cyclic voltammetry. The reproducibility results are listed in Table 2. The relative standard deviation (RSD) was 0.45%, indicating that the TiO_2_–ErGO–GCE exhibited good reproducibility for tartrazine detection.

### 3.6. Real Sample Detection

SDLSV shows high resolution and sensitivity in electrochemical analyses, thus it is widely applied in food additives detection. In this section, this method was used to analyze carbonated beverage samples. These carbonated beverage samples at various concentration were measured by using SDLSV under the optimal conditions. A shown inn Table 3, the concentration of tartrazine detected in samples 1–3 were 0.2, 2.24, and 4.26, respectively, which were well consistent with the standard values. Moreover, the corresponding RSD was 1.05–2.32%, and the recovery rate of the samples 2 and 3 was 112.0% and 106.5%, respectively. These results indicate that the TiO_2_–ErGO-GCE could be an efficient system for tartrazine detection in carbonate beverage samples.

## 4. Conclusions

In summary, a TiO_2_–ErGO–GCE was successfully fabricated by hydrothermal and electrochemical reduction and it was used for practical tartrazine detection. After electrochemical reduction, the TiO_2_ NPs were coated by ErGO. The oxidation peak current on the TiO_2_–ErGO–GCE increased to 26.98 μA, which was 18 times higher than that on the bare GCE. Moreover, the electrochemical results revealed that the electrochemical oxidation of tartrazine is an adsorption-controlled process with one electron and one proton. A wide linear range (from 2 × 10^−8^ mol/L to 2 × 10^−5^ mol/L) and a low detection limit (S/N = 3) of 8.0 × 10^−9^ mol/L were also obtained with the TiO_2_–ErGO–GCE. Finally, in practice, the TiO_2_–ErGO–GCE also showed a good detection sensitivity in the detection of tartrazine in a carbonated beverage. This detection system shows great application prospects for the sensitive detection of food additives in real samples, due to its prominent advantages including facile fabrication, rapid response, good selectivity, low detection limit, and wide linear range of detection. 

## Figures and Tables

**Table 1:** The influence of different interferents on the detection ability of tartrazine by TiO_2_–ErGO–GCE.

Interferents	Peak Currents *i_pa_* (μA)
Without interferents (10 μmol/L of tartrazine)	36.73
Glucose (100-times concentration)	36.80
Benzoic acid (100-times concentration)	36.30
Citric acid (100-times concentration)	36.27
Na^+^ (100-times concentration)	36.69
K^+^ (100-times concentration)	36.75
Fe^3+^ (100-times concentration)	36.71
Amaranth (10-times concentration)	36.54
Sunset yellow (10-times concentration)	36.60

**Table 2:** The reproducibility of TiO_2_–ErGO–GCE determination of tartrazine.

No.	1	2	3	4	5	6	7
*i_pa_*/μA	36.82	37.52	36.99	37.20	37.00	36.95	36.98
Average value/μA	37.06						
RSD/%	0.45						

**Table 3:** The results of the determination of tartrazine in a soft drink at different adding concentrations (*n* = 3).

No.	Added/(μmol/L)	Total Found/(μmol/L)	Recovery (%)	RSD/(%)
1	—	0.20	—	2.32
2	2	2.24	112.0	1.05
3	4	4.26	106.5	1.50
